# A Versatile Approach for the Synthesis of Antimicrobial Polymer Brushes on Natural Rubber/Graphene Oxide Composite Films via Surface-Initiated Atom-Transfer Radical Polymerization

**DOI:** 10.3390/molecules29040913

**Published:** 2024-02-19

**Authors:** Wenya Zhu, Bangsen Li, Jinrui Liu, Shishu Sun, Yan Zhang, Dashuai Zhang, Chen Li, Tianyi Sun, Huaide Qin, Jianjun Shi, Zaifeng Shi

**Affiliations:** 1 Collage of Chemistry and Chemical Engineering, Hainan Normal University, Haikou 571158, China; 18217902632@163.com (W.Z.); bangsenli@163.com (B.L.); jinruiliu1996@163.com (J.L.); zy936457505@163.com (Y.Z.); lichenzi720@163.com (C.L.); tianyi870328@163.com (T.S.); 2 Key Laboratory of Water Pollution Treatment and Resource Reuse of Hainan Province, Haikou 571158, China; 3 Haikou Key Laboratory of Water Environmental Pollution Control, Haikou 571158, China; 4 Rubber Research Institute, Chinese Academy of Tropical Agricultural Sciences, Haikou 571101, China; qinhuaide@yeah.net

**Keywords:** natural rubber, NR/GO composite film, atom transfer radical polymerization, antimicrobial materials

## Abstract

A simple strategy was adopted for the preparation of an antimicrobial natural rubber/graphene oxide (NR/GO) composite film modified through the use of zwitterionic polymer brushes. An NR/GO composite film with antibacterial properties was prepared using a water-based solution-casting method. The composited GO was dispersed uniformly in the NR matrix and compensated for mechanical loss in the process of modification. Based on the high bromination activity of α–H in the structure of *cis*-polyisoprene, the composite films were brominated on the surface through the use of *N*-bromosuccinimide (NBS) under the irradiation of a 40 W tungsten lamp. Polymerization was carried out on the brominated films using sulfobetaine methacrylate (SBMA) as a monomer via surface-initiated atom transfer radical polymerization (SI-ATRP). The NR/GO composite films modified using polymer brushes (PSBMAs) exhibited 99.99% antimicrobial activity for resistance to Escherichia coli and Staphylococcus aureus. A novel polymer modification strategy for NR composite materials was established effectively, and the enhanced antimicrobial properties expand the application prospects in the medical field.

## 1. Introduction

Natural rubber (NR) is an essential material in engineering applications. It is widely used in medical fields due to its high wear and barrier property levels; it is used in supplies such as medical gloves [1,2], condoms [3,4,5], and catheters [6]. The preventative properties of natural rubber latex (NRL) can be applied to manufacture products that isolate pathogens, and NRL is one of the best natural raw materials for biomedical products. However, protein adsorbed on the surface of NR [7] makes it easy for bacteria to contaminate the surface of the material. The adsorption of non-specific proteins, such as via the blockage of the filter membrane during a biological separation procedure or blood clotting in an infusion catheter, can cause potential harm. The initial formation of biological scale and blood clots takes place in the stage of the rapid adsorption of proteins at the molecular level on the surface of an NR material. The development of anti-protein-adsorption NR materials can prevent bacterial adsorption and other biological fouling phenomena in the procedure of the adsorption of biological macromolecules at the solid–liquid interface. Various pathogenic bacteria also harm the health of patients receiving medical treatment. Although the antibacterial properties of natural rubber can be realized through the use of doping and blending certain fillers [8,9,10], an active surface is difficult to maintain in the long term. The modification of antibacterial active molecules on the surface of a substrate can confer long-term antibacterial activity, but there are few studies on the fixation of antibacterial active molecules on the surface of an NR substrate [11]. Grafting antibacterial molecules [12,13] onto the surface of NR directly is a potential way of preparing NRL medical products, but it is difficult to obtain a high-density antibacterial layer on the surface of NR.

Effectively, cationic or zwitterionic macromolecular materials will endow polymers with antibacterial and resistant properties for the modification of various materials through the use of “grafting-from” and “grafting-to” approaches. The hydrated layers in zwitterionic materials can bind tightly through electrostatic interactions, resulting in these materials being able to effectively resist the adhesion of dirt agents [14]. The amphiphilic structures of synthetic polymers can destroy the cell membrane, rupturing the transmembrane potential and causing leakage of cytoplasmic contents, eventually leading to cell death [15]. The “grafting-to” method refers to the synthesis of the main chain and the functionalization of the side chain in a polymer structure. The design of specific properties in a polymer’s structure is based on the functionalization of the side chain. Ying-Nien Chou et al. [16] exploited a zwitterionic polymer, ploy(glycidyl methacrylate-*co*-sulfobetaine methacrylate), which was grafted onto the surfaces of versatile biomaterials depending on the base-induced ring-opening reaction between epoxied and hydroxyl groups. Qin Cao et al. [17] treated an organosilicon film using vacuum ultraviolet radiation and grafted zwitterionic sulfobetaine layers with a cross-linked and comb-like structure covalently to silicone thin films. The obtained silicone films showed better hydrophilicity, antibacterial properties, and biocompatibility. Although the operation of the “grafting-to” approach for the preparation of antibacterial substrates is simple and convenient, a low polymer coverage rate is achieved due to steric hindrance. The “grafting-from” approach requires immobilizing initiators on the surface of the substrate as active sites, and the propagated polymer chains are initiated from these sites. The advantage of this approach lies in the low steric hindrance for polymer growth and the high coverage rate of the grafted polymer.

Strategies of controllable polymerization, such as atom transfer radical polymerization (ATRP) [18,19,20,21,22], reversible addition–fragmentation chain transfer polymerization (RAFT) [23,24,25,26], ring-opening polymerization (ROP) [27,28], and ring-opening metathesis polymerization (ROMP) [29,30,31], are available “grafting-from” methods for surface-initiated polymerization that can be used in the modification of various substrates. Qian Ye et al. [32] introduced a novel surface-initiated ring-opening metathesis polymerization (SI-ROMP) to prepare high-thickness zwitterionic polymer brushes functionalized through the use of imidazolium salts. The obtained polymer brushes not only have effective resistance against bovine serum albumin and algal adhesion but also antibacterial activity against Escherichia coli (*E. coli*) and Staphylococcus aureus (*S. aureus*). The homogeneous polymer brushes employing sulfobetaine methacrylate as a monomer were covered onto gold surfaces via the surface-initiated ATRP (SI-ATRP) method, and super-low-fouling gold plates modified with PSBMA brushes were achieved to resist marine fouling while being environmentally friendly [33]. ATRP has become a common method for surface modification with polymer brushes and relies on a reversible activation–deactivation balance between transition metal complexes or metal-free catalysts and the halogenated alkyl ends of dormant polymer chains to produce free radicals that can multiply in the presence of monomers [34].

**Figure 1:**
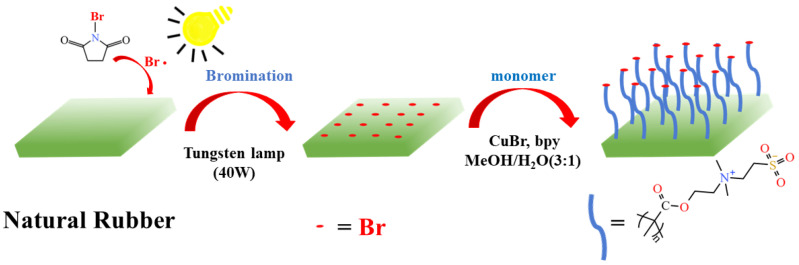
Radical bromination of NR/GO film and SI-ATRP of methacrylate monomers.

Various methods for the preparation of brominated NR (NR-Br) in solution [35] or emulsion [36,37] have been reported. Recently, a strategy involving the use of NR-Br as a macromolecular initiator to graft styrene polymer in emulsion was adopted [38]. However, this strategy does not apply to the targeted modification of NR. The targeted modification of the surface avoids the complex chemical process of functionalization in the internal NR substrate, thus, preserving the physical and mechanical properties of NR materials. Interestingly, the bromination of NR films can form active sites for initiators on the surface, and further modification based on polymer brushes changes the surface properties through the “grafting from” approach. Based on the high bromination activity of α–H in the structure of *cis*-polyisoprene, the preparation of the macromolecular initiator (Allyl bromide structure) [39] can be completed in one step, avoiding the complex anchoring process of the initiation site. In this bottom-up grafting strategy, the polymer chains are grown from the substrate and modified by functional groups that can initiate the polymerization reaction, and surface polymer brushes with high grafting density can be obtained. Herein, the NR/GO composite films were prepared using the water-based solution-casting method. The introduction of GO can compensate for mechanical loss in the modification process. *N*-bromosuccinimide (NBS) was adopted to brominate the NR/GO films directly under the condition of 40 W tungsten lamp irradiation. The antibacterial polymer brushes using SBMA as a monomer were initiated on the NR/GO surface, and the process is shown in Figure 1. This novel and practical method imparts obvious improvements to the antimicrobial properties of NR/GO films.

## 2. Results and Discussion

### 2.1. Characterization of NR/GO Film, NR/GO-Br Film, and NR/GO-PSBMA Film

**Figure 2:**
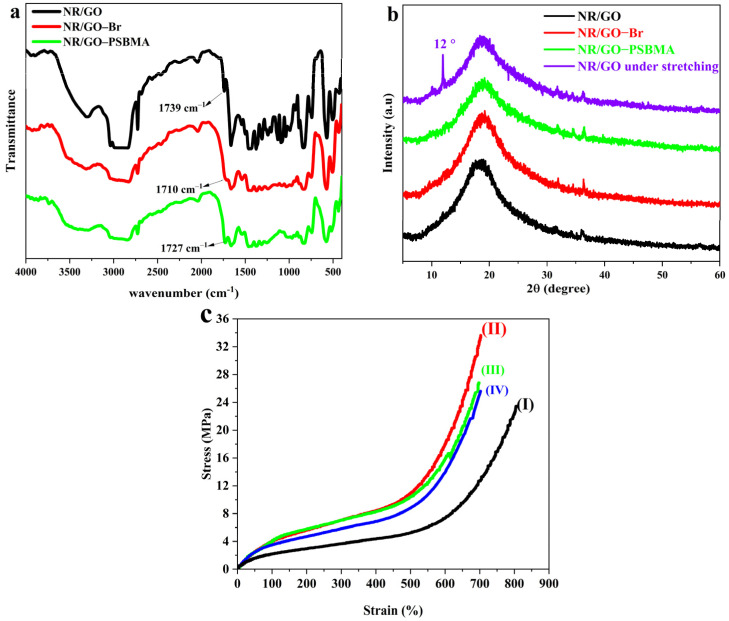
(**a**) FTIR spectra of NR/GO film, NR/GO-Br film, and NR/GO-PSBMA film. (**b**) XRD patterns of NR/GO film, NR/GO-Br film, NR/GO-PSBMA film and NR/GO film at stretching. (**c**) Stress–strain curves of NR-S films (I), NR/GO film (II), NR/GO-Br film (III), and NR/GO-PSBMA film (IV).

The chemical structure of the NR/GO film, NR/GO-Br film, and NR/GO-PSBMA film were characterized through the use of FTIR spectroscopy (Figure 2a). The spectrum of the NR/GO film had a wide absorption band at 2830–3000 cm^−1^, which was attributed to the aliphatic C-H stretching of NR’s main chain structure. The peak at 1662 cm^−1^ was the stretching vibration absorption of –C=C–. The peak at 1739 cm^−1^ may account for the protein, fatty acid, and sugar in the NR substrate. After the modification of bromination, the new stretching vibration peak of C–Br should appear at 515–690 cm^−1^, but it was covered by the out-of-plane bending vibration of the C–H bond in the NR structure. The peak of C=O appeared at 1710 cm^−1^ due to the small partial oxidation of carbonyl groups from GO, or C=C being converted to C=O of NR substrate under the etching action of NBS. After grafting the polymer brushes, a new, obvious peak around 1727 cm^−1^ attributed to C=O in the ester group of PSBMA was observed, indicating that the PSBMA was successfully grafted to the surface of the NR/GO composite films using the SI-ATRP method.

To analyze the dispersion of nanoscale GO sheets in the structure of the composite films, XRD characterization was utilized, and the results are shown in Figure 2b. The NR/GO composite films showed a broad diffraction peak around 20°, which is attributed to the non-crystalline structure of NR. The weak diffraction peaks between 30° and 40° belonged to the ZnO residual relating to the suspension of the vulcanizing agent. The characteristic absorption peak of GO was difficult to observe, manifesting in GO sheets that are evenly dispersed in the NR matrix. NR easily crystallizes during the stretching treatment. From the XRD pattern of NR/GO under stretching action, the diffraction peak near 12° was assigned to the (200) plane reflections of NR [40]. The composited GO was beneficial to stress transfer and strain-induced crystallization, both of which improved the modulus of the composites [41]. Because of the increased boundary or interface area between the nano-filler and the NR matrix based on the large specific surface area of the nano-filler [42], the homodisperse of GO was a crucial factor in improving the mechanical properties of the modified NR composite films. 

Tensile tests were adopted to estimate the effects of surface bromination and SI-ATRP on the physical and mechanical properties of the NR/GO films (sample dimension, 2 mm × 60 mm × 0.5 mm). The mechanical properties and corresponding stress–strain curves of NR/GO composite film are shown in Table 1 and Figure 2c, respectively. Because GO can form a uniform network in the polymer matrix [42], the modulus and tensile strength (at 100% and 300% MPa) of the NR/GO film were improved, and the tensile strength values increased by 102%, 95%, and 43%, respectively. The elongation at the break had a slight decrease from 796% to 700% compared with vulcanized NR (NR-S) without the addition of nano GO. After the surface modification, the elongations at the break between NR/GO-Br (690%) and NR/GO-PSBMA (705%) were quite close to each other, but the corresponding tensile strength decreased by 20% and 27%, respectively. The decreased tensile strength could be caused by the attack of bromine radicals from NBS and free radicals from the SI-ATRP process on the π electron in the main chain of NR. After the polymer modification, the mechanical properties of NR/GO-SBMA remained, and the composited GO compensated for the mechanical loss in the process of modification.

**Figure 3:**
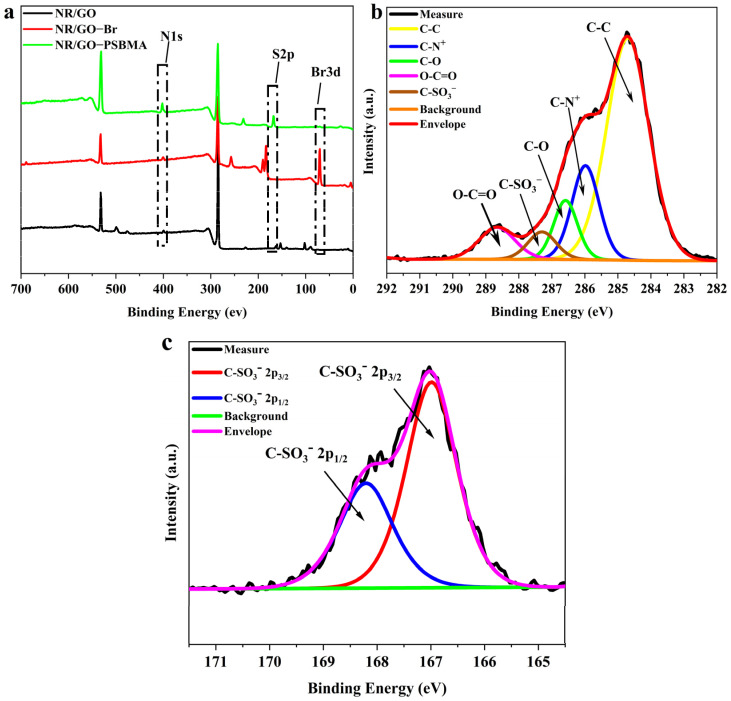
XPS spectra of (**a**) NR/GO film, NR/GO-Br film, and NR/GO-PSBMA film, (**b**) C1s and (**c**) S2p core-level spectra of the NR/GO-PSBMA.

NBS, as a mild active bromine reagent, has high selectivity and fewer side reactions, and bromination is carried out under moderate conditions [43,44,45]. A 40 W visible tungsten lamp was used as the light source initiator to avoid NR degradation caused by UV irradiation. The surface chemical composition of NR/GO, NR/GO-Br, and NR/GO-PSBMA films was characterized using XPS before and after modification (Figure 3a). The peaks at 285 eV (C_1s_) and 531 eV (O_1s_) were assigned to the characteristic signals of NR and GO. A small peak at 400 eV (N_1s_) could be caused by the protein derived from NRL. After the bromination treatment with NBS, an obvious peak appeared at 70.08 eV (Br_3d_), representing the brominated α–H of *cis*-polyisoprene in the structure NR/GO-Br film. The characteristic signals from the grafted PSBMA appeared at 168 eV (S_2p_) and 402 eV (N_1s_). Furthermore, the corresponding atomic percentages of C, O, N, Br, and S elements are summarized in Table 2. The C/O atomic ratio value (~2.93) and C/N atomic ratio value (~13.59) of NR/GO-PSBMA composite films agreed with the calculated values of neat SBMA (C/O atomic ratio value of ~2.19 and C/N atomic ratio value of ~10.99). The ratio of S_2p_/N_1s_ value was close to 1:1 with the increase in polymerization time, which was consistent with the literature reports [46]. The results revealed that PSBMA, in the form of a dense layer, was covalently grafted onto the NR/GO composite film surface. The decreased signal of the Br element could be caused by the termination reaction of active chains in the ATRP process, and it led to a few bromine atoms existing at the end of the chains. The only source of the S element was from the polymer brushes of SBMA, and the grafting ratio (GR) of SBMA was calculated according to Equation (1) [47]:

(1) $$\mathrm{Gr}=\frac{\mathrm{NR}/\mathrm{GO}-P{\mathrm{SBMA}}_{S2P}}{{\mathrm{neat}\;\mathrm{SBMA}}_{S2P}}\times 100\%$$

where NR/GO-PSBMA_S2P_ is the percentage of sulfur atoms on the NR/GO-PSBMA film surface measured using XPS; and neat SBMA_S2P_ is the sulfur atomic percentage of SBMA when the NR/GO-PSBMA film surface is completely covered with PSBMA.

The core-level binding energies of the PSBMA-modified composite film were determined by examining the C1 slight-splitting spectra (Figure 3b). The C1s peak was split into five components containing CC (284.7 eV), C–N^+^ (285.98 eV), C–O (286.58 eV), C–SO_3_^−^ (287.3 eV), and O–C=O (288.68 eV). In the S2p core-level spectrum, the strong spin-orbital split doublet signal from polymer brushes can be fitted to the components of the S2p3/2 and S2p1/2 peaks at their respective binding energies of 167 and 168.2 eV (Figure 3c) [48]. The received results from integrations of deconvoluted peaks (C–C: 60.36%, C–N^+^: 17.80%, C–O: 9.22%, C–SO_3_^−^: 5.10%, and O–C=O: 7.52%) were roughly identical to the theoretical values of neat PSBMA brushes as shown in Table 3. This also indicated that the layer of PSBMA grafted on the NR/GO film surface was relatively dense.

**Figure 4:**
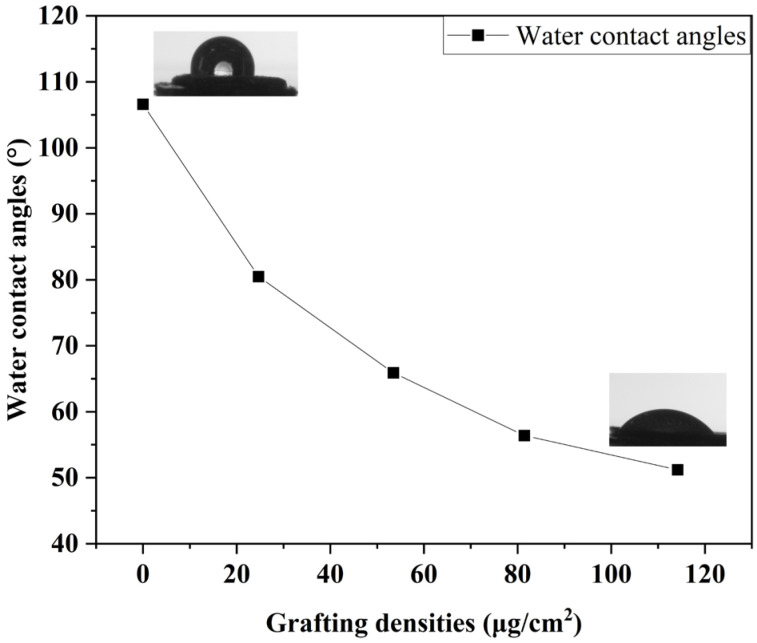
Contact angle on the NR/GO-SBMA film surface with different grafting densities.

Zwitterionic polymers are extremely hydrophilic due to electrostatic interactions [34]. The surface properties of PSBMA brush-grafted NR/GO-PSBMA films can be evaluated via static water contact angle (WCA) measurements. The WCA of the NR/GO composite film had a high contact angle of 118.8°, while the WCA of the NR/GO-Br film reduced to 106.6° owing to the introduction of Br atoms. When the Gr was equal to 79.50%, the polymer brush with a higher density had much more hydrophilicity (WCA = 35.6°), as shown in Table 2 and Figure 4.

### 2.2. Antibacterial Activity of NR/GO Films

**Figure 5:**
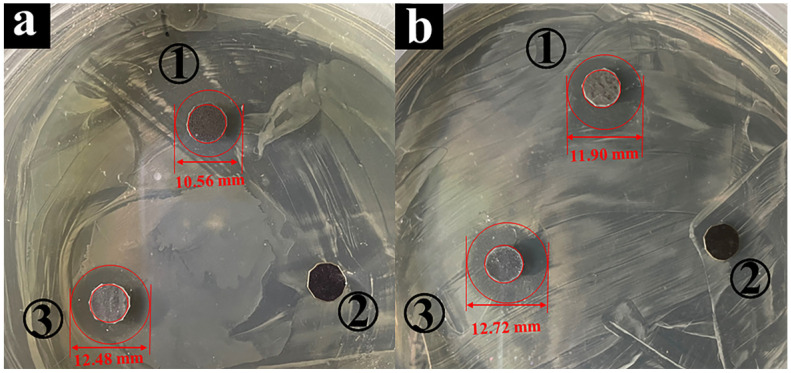
Antibacterial activity assay by disc diffusion against (**a**) *E. coli* and (**b**) *S. aureus* provided by (1) non-brominated NR/GO film exposed to ATRP conditions (CuBr, bpy, SBMA, and MeOH-H_2_O), (2) NR/GO film containing 5 μg ciprofloxacin, and (3) NR/GO-PSBMA film.

The ability to resist bacterial adhesion or biofilm formation depends on certain factors for the NR/GO composite film, such as surface properties, bacterial strength, growth medium, and temperature [49]. The zwitterionic groups of the modified polymer brushes on the substrate surface have high resistance to non-specific protein adsorption, bacterial adhesion, and biofilm formation due to electrostatic-induced hydration [49,50,51]. The cationic portion of the zwitterionic groups interacts with negatively charged bacterial cell membranes to result in the effect of cell lysis [52,53,54]. Gram-negative bacterium *E. coli* and Gram-positive bacterium *S. aureus* are common and representative infection strains [55], which are used to evaluate the antibacterial effect of the NR/GO and NR/GO-PSBMA films. Antibacterial activity was determined through the use of the disc diffusion assay of NR/GO films and NR/GO-PSBMA films (Figure 5). The inhibition zone against *E. coli* was not observed in the sample of NR/GO film (a_2_). In contrast, the NR/GO-PSBMA films (a_3_) showed a clear inhibition zone (d = 12.48 ± 0.21 cm), and *E. coli* could not grow in this zone. For *S. aureus*, the NR/GO-PSBMA (b_3_) films also showed a clear inhibition zone (d = 12.72 ± 0.25 cm), and the inhibition zone was not observed in the sample of NR/GO film (b_2_). The PSBMA brush-modified NR/GO composite films had high grafting density and excellent antibacterial activity based on the “grafting-from” method. 

**Figure 6:**
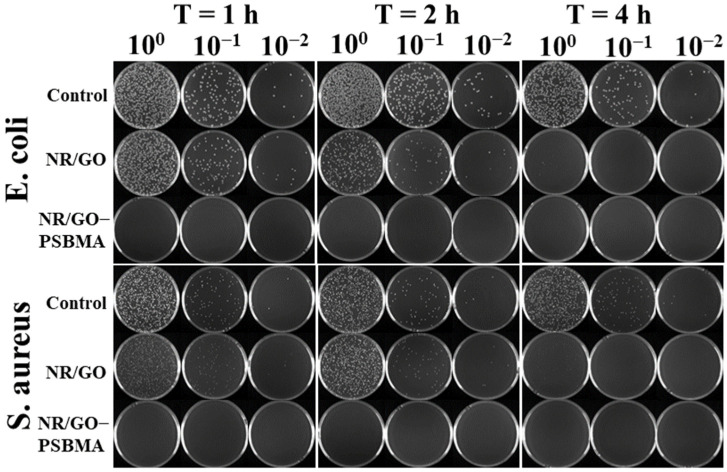
Antimicrobial activity assessment against *E. coli* and *S. aureus* of the films by coculture and plate coating count test. Cell viability was determined by spotting samples of serial 1:10 dilutions of the bacterial suspensions at different exposition times.

The bacterial culture medium of *E. coli* was diluted to 10^6^ CFU/mL with PBS solution, and 50 μL of the diluted solution was spread on the surface of the sterilized samples. After elution, dilution, and incubation, the plating of the resulting cell suspension on agar is shown in Figure 6. The antibacterial rates of the NR/GO films (non-brominated NR/GO film exposed to ATRP conditions) against *E. coli* after the exposure for 1, 2 and 4 h were 17.68 ± 2.10%, 62.88 ± 2.42%, and 95.79 ± 2.65%, respectively. The antibacterial efficiency of the NR/GO films (non-brominated NR/GO film exposed to ATRP conditions) against *S. aureus* after exposure for 1, 2, and 4 h was 0%, 8.56 ± 1.85%, and 81.21 ± 3.35%, respectively. The antibacterial activity of non-brominated NR/GO films may be caused by exposure to residual SBMA and copper ions [56] under ATRP conditions and the action of GO [57] in the films. Therefore, it is necessary to choose a non-brominated NR/GO film for the comparison to verify the excellent antimicrobial properties of NR/GO-PSBMA. Interestingly, the NR/GO-PSBMA film completely killed *E. coli* and *S. aureus* under different exposure times. The antibacterial efficiency of the composite films was up to 99.99% in 1 h, indicating that the grafted zwitterionic PSBMA brushes improved the bactericidal effect of the NR/GO composite films. The high antibacterial effect is due to the fact that the PSBMA hydration layers are bound together tightly through electrostatic interactions, which makes these materials more effective against non-specific protein adsorption and bacterial adhesion resistance [14]. Moreover, the cationic part of the zwitterionic group can also interact with the negatively charged bacterial cell membrane to lyse the cell [46]. PSBMA can bind to a single regulator, such as protein, on the bacterial membrane, indirectly inhibiting the biosynthesis of macromolecules, such as tRNA and RNA [58].

**Figure 7:**
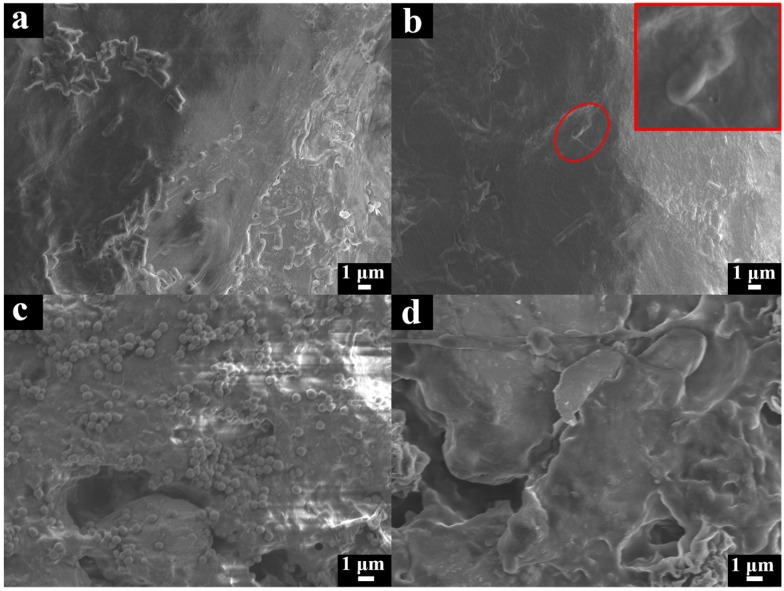
SEM images of films: (**a**) non-brominated NR/GO film exposed to ATRP conditions (CuBr, bpy, SBMA, and MeOH-H_2_O) and (**b**) NR/GO-PSBMA film after *E. coli* bacterial attachment, the red square is a magnified image of the red circle; (**c**) non-brominated NR/GO film exposed to ATRP conditions (CuBr, bpy, SBMA, and MeOH-H_2_O) and (**d**) NR/GO-PSBMA film after *S. aureus* bacterial attachment.

There was a mountain of *E. coli* (Figure 7a) and *S. aureus* (Figure 7c) attached to the surface of the NR film in the SEM photos. On the contrary, the surface of the NR/GO-PSBMA films showed fewer *E. coli* (Figure 7b) and *S. aureus* (Figure 7d) attached to the surface because the PSBMA exhibited high resistance to bacterial adhesion and prevented the formation of biofilm [49]. In addition, the morphology of *E. coli* bacteria (Figure 7b) attached to the surface of NR-PSBMA was irregular and underwent partial collapse. The potential mechanism of this phenomenon was that the PSBMA polymer induced cytotoxicity, cell growth arrest, and cell death by binding to bacterial cell membranes to produce reactive oxygen species (ROS) [58,59,60].

**Figure 8:**
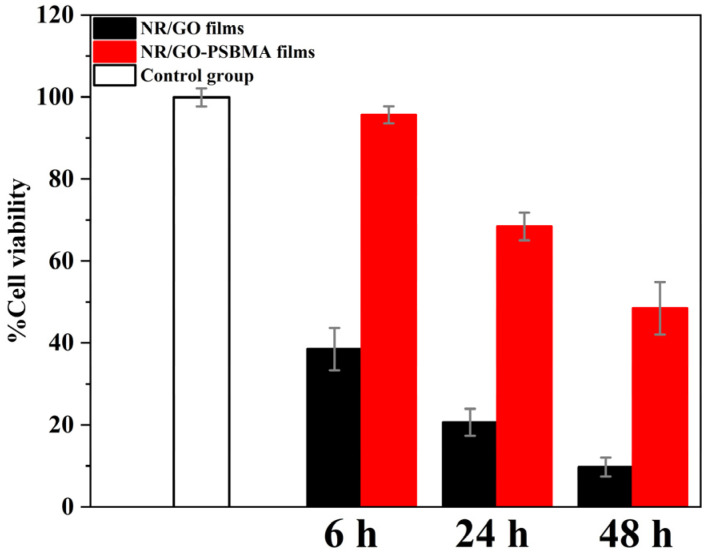
Treatment of MCF-7 cells with NR/GO films and NR/GO-PSBMA films.

The standard MTT assay was employed for the further verification of cytotoxicity, as shown in Figure 8. The results displayed extremely low toxicity (95.64% survival rate) in comparison to NR/GO films (38.49% survival rate) against MCF-7 cells after incubation for 6 h. The cell survival rate remained above 68.45% after co-culture with the NR/GO-PSBMA films for 24 h. The grafted polymer led to an approximate cell death of 51.55% after the incubation time was prolonged to 48 h, and this result was still lower than 90.28% of NR/GO films. The results of the cytotoxicity test towards MCF-7 cells indicated that the toxicity of NR/GO-PSBMA films to mammalian cells was less than that of NR/GO films within 48 h. The lower cytotoxicity of NR/GO-PSBMA films compared with NR/GO films was attributed to the good biocompatibility of PSBMA with high surface graft density. This approach could be a potential candidate for the NRL-based medical products used in the medical field. Further reducing the toxicity of NR/GO-PSBMA membranes to cells in subsequent studies, such as the use of green and environmentally friendly metal-free organic photocatalysts [61], will give the method better application prospects.

## 3. Experimental Section

### 3.1. Materials

Zinc oxide (ZnO, 99%), zinc diethyldithiocarbamate (ZDC, 98%), *N*-bromosuccinimide (NBS, 99%), CuBr (99%), 2,2′-bipyridine (bpy, 99%), phosphate-buffered saline (PBS, pH = 7.4), MTT (3-(4,5-dimethylthiazol-2-yl)-2,5-diphenyltetrazolium bromide), and [2-(methacryloyloxy) ethyl] dimethyl-(3-sulfopropyl) ammonium (≥97%) were purchased from Shanghai Aladdin Biochemical Technology Co., Ltd. (Shanghai, China). Graphene oxide (diameter: 0.5–5 mm; thickness: 0.8–1.2 nm) and KOH (95%) were purchased from Shanghai Maclin Biochemical Technology Co., Ltd. (Shanghai, China). NR latex was provided by the Rubber Research Institute of the Chinese Academy of Tropical Agricultural Sciences (Haikou, China). All other reagents and solvents were purchased from commercial suppliers and were used directly without further purification.

### 3.2. Preparation of Brominated NR/GO Composite Films

Sulfur, ZnO, KOH, ZDC, and GO were added to deionized water and treated with ultrasonic dispersion for 30 min. The obtained suspension was added to the NR latex (NRL) and stirred until smooth to prepare a homogenous mixture at 60 °C. Then, the NRL/GO mixture was poured into a Petri dish. After the evaporation of water at room temperature overnight, the NR/GO composite films were carefully peeled off from the Petri dish and subjected to vulcanization using the oven (electric blast drying oven, GZX-9140MBE, Boxun, Shanghai, China) at 90 °C for 2 h. The thickness of the received NR/GO composite films was about 0.5 mm. The composition of rubber ingredients and fillers was as follows (in phr, where phr meant parts per 100 parts of NRL): S (1 phr), ZnO (0.8 phr), KOH (0.3 phr), ZDC (0.4 phr), and GO (0.5 phr).

### 3.3. Preparation of Brominated NR/GO-Br Composite Films

The NR/GO composite films were added to a solution of NBS (20 mL, 14 mg/mL solution in deionized H_2_O), and the bromination occurred under the irradiation of a 40 W tungsten lamp. The reaction solution was replaced by fresh NBS (20 mL) solution every 8 h, and the reaction was carried out for 24 h at room temperature. The obtained NR/GO-Br films were washed three times with deionized H_2_O and MeOH, respectively, and dried under a stream of N_2_.

### 3.4. Preparation of PSBMA-Modified NR/GO Composite Films (NR/GO-PSBMA) Using SI-ATRP Methods

Transition metal complexes produced by CuBr and bpy can trigger the production of free radicals from halogenated alkyl ends of dormancy initiators that can multiply in the presence of monomers. Classical ATRP allows the presence of water, but oxygen will quench the free radicals, so the reaction takes place under N_2_ protection. SI-ATRP was performed according to the reported methods [46]. The specific polymerization process was as follows: 2,2′-Bipyridine (bpy, 12 mg, 0.075 mmol) was added to a solution of methacrylate monomer (2.4 g, 8.6 mmol) in 6 mL of a mixture of MeOH and H_2_O (*v*:*v* = 3:1) in a 50 mL Schlenk flask. The reaction mixture was degassed using six freeze–pump–thaw cycles and backfilled with N_2_. The brominated NR/GO films were placed in a round-bottom flask, and CuBr (4 mg, 0.03 mmol) was added. The degassed bpy and methacrylate monomer solution (2 mL) was added to the flask, and the ATRP reaction was initiated on the surface of NR/GO-Br in a shaker (full temperature oscillating incubator, HZQ-F160, Baidian, Shanghai, China). A temperature of 37 °C was maintained during the polymerization, and the grafting process needed 2–8 h. The modified composite films were taken out from the flask and rinsed thrice with H_2_O and MeOH. After vacuum drying at 40 °C to a constant weight, the grafting density (GD, μg/cm^2^) was calculated using the following equation:

(2) $$\mathrm{GD}=\frac{W_{1}-W_{0}}{A_{0}}$$

where W_0_ and W_1_ are the mass of the NR/GO-Br film and NR/GO-PSBMA film. A_0_ represents the area of the films.

### 3.5. Evaluation of Antibacterial Properties

#### 3.5.1. Antibacterial Activity by Disc Diffusion Assay

The test of the antibacterial properties was performed using the disk diffusion method. The bacterial phosphate-buffered saline (PBS, pH = 7.4) solution containing 10^6^ CFU/mL was spread over the Mueller–Hinton Broth. The composite films (*Φ* = 6 mm) were placed on the agar medium after being sterilized under an ultraviolet lamp for 30 min. It was incubated in an incubator (biochemical incubator, LRH-80, Juchuang, Qingdao, China), and the inhibition areas were observed after 8 h. The NR/GO containing 5 μg ciprofloxacin was used as the positive control, and NR/GO was used as the negative control.

#### 3.5.2. Antimicrobial Activity Assessment of NR Films by Co-Culture and Plate Coating Count Test

Then, 3 mL of Luria–Bertani (LB) liquid medium was added into 12 mL bacterial culture tubes. The bacteria were inoculated in the LB broth overnight in a constant-temperature oscillator (37 °C, 200 rpm). The composite films (*Φ* = 6 mm) were sterilized under ultraviolet lamp irradiation for 30 min and then placed into disposable Petri dishes. The bacterial culture medium of bacteria was diluted to 10^6^ CFU/mL with PBS solution, and 50 μL of the diluted solution was spread on the surface of sterilized samples. The culturing process was undertaken at a constant temperature (37 °C) for 1 h, 2 h, and 4 h. The suspension of bacteria was eluted continuously 10 times with 950 μL sterile PBS solution, and 100 μL diluent was coated on LB solid medium evenly. After incubation for 18 h, the number of colonies was recorded. The percentage of the antibacterial rate was calculated using the following equation:

(3) $$\mathrm{Antibacterial}\;\mathrm{rate}=1-\frac{C_{0}-C_{1}}{C_{0}}\times 100\%$$

where C_1_ = experimental group bacterial concentration and C_0_ = control group bacterial concentration

#### 3.5.3. The Adhesion of Bacteria on the Composite Films Was Evaluated Using Scanning Electron Microscopy

The composite films were taken out from disposable Petri dishes after co-culture for 1 h. The composite films were fixed with 2.5% glutaraldehyde for 6 h and then washed 3 times with sodium phosphate buffer (pH 7.4). The films were dehydrated with 25%, 50%, 75%, and 100% ethanol and vacuum dried at 37 °C overnight. The samples were analyzed using field emission scanning electron microscopy (SEM, JEOL, Tokyo, Japan).

### 3.6. Measurement of Toxicity toward Mammalian Cells

The density of the MCF-7 cell suspensions was adjusted to 2 × 10^4^ cells/well. The suspensions were placed on a 96-well plate (90 μL/well) and incubated at 37 °C for 24 h. The edge holes were filled with the same amount of sterile PBS to avoid the edge effect. The polymer films were incubated in the wells of cells for 6 h, 24 h, and 48 h, respectively. MTT (3-(4,5-dimethylthiazol-2-yl)-2,5-diphenyltetrazolium bromide, 10 μL/well) was added, and the solutions were incubated at 37 °C for approximately 4 h. The medium was carefully aspirated with a multi-channel straw, and then 100 μL DMSO was added to each well. The plate was placed on the rocker and shaken for 10 min to dissolve the MTT-formazan completely. The absorbance value was detected at 570 nm. Cell viability was measured as the fraction of absorbance relative to the control hole.

### 3.7. Characterizations

FTIR spectra were recorded on a Thermo Scientific Nicolet 6700 spectrometer (Waltham, MA, USA) with a 4 cm^−1^ resolution under a wavenumber range of 4000–400 cm^−1^ and 32 average scans at room temperature. The samples were treated using the KBr pellet pressing method. XRD analysis was examined using an Ultima IV X-ray diffractometer (Rigaku, Tokyo, Japan) in the 2θ range from 5 to 60° with a scan rate of 5°/min and a monochromatic Cu Ka source (λ = 0.154056 nm). The XPS spectra were analyzed using a Thermo Scientific K-Alpha electron spectrometer with an Al-Kα source. Scanning electron microscopy (SEM) images were captured on a JSM-7100F instrument (JEOL, Tokyo, Japan) under an acceleration voltage of 5 kV. Tensile tests were conducted on a universal testing instrument (WDW-1KN; Jinan Yinuo Century Test Instrument Co., Ltd., Jinan China) with 500 mm/min of tensile speed and 2 mm× 60 mm × 0.5 mm of sample dimension selected. The tensile properties of each composite were tested three times.

## 4. Conclusions

In conclusion, the NR/GO composite film was modified through the use of polymer brushes of PSBMA on the brominated surface combined with SI-ATRP graft modification. The surface modification method avoided complex chemical processes and retained the good physical and mechanical properties of the NR composite material. The anchor point of the trigger site can be completed in one step, avoiding the complex initiation site anchoring process. Subsequently, the PSBMA polymer brushes grew on the surface of the NR/GO films with a high grafting rate through the “grafting from” approach, and the modified composite films had better antibacterial activity compared with neat NR/GO films. Therefore, this approach provides abundant possibilities for surface modifications of NR with antimicrobial polymer brushes and acceptable potential in industrial and medical applications. Moreover, this method also provides abundant possibilities for the surface grafting of NR to various functional polymers.

## Figures and Tables

**Table 1:** Mechanical properties of NR/GO composite films.

Samples	NR	NR/GO	NR/GO-Br	NR/GO-PSBMA
Modulus at 100% (MPa)	2.05 ± 0.26	4.15 ± 0.35	4.23 ± 0.25	3.65 ± 0.18
Modulus at 300% (MPa)	3.55 ± 0.30	6.95 ± 0.41	6.96 ± 0.33	5.63 ± 0.26
Tensile Strength (MPa)	23.36 ± 0.35	33.39 ± 0.56	26.67 ± 0.39	24.50 ± 0.23
Elongation at Break (%)	796 ± 15	700 ± 16	690 ± 12	705 ± 16

**Table 2:** Surface composition of NR/GO composite films with different polymerization times from XPS.

Sample	C_1s_ (%)	N_1s_ (%)	O_1s_ (%)	Br_3d_ (%)	S_2p_ (%)	GD ^a^ (µg/cm^2^)	Gr ^b^	WCA (°)
NR/GO	86.68	0.51	12.81	-	-	-	-	118.8
NR/GO-Br	74.41	1.67	13.25	10.67	-	-	-	106.6
NR/GO-PSBMA_2h_	73.14	2.24	16.58	5.92	2.12	24.68	38.13	80.5
NR/GO-PSBMA_4h_	72.76	2.92	18.18	3.79	2.35	53.45	42.45	65.9
NR/GO-PSBMA_6h_	72.17	4.09	19.34	1.01	3.39	81.46	60.97	56.4
NR/GO-PSBMA_8h_	67.43	4.96	22.99	0.20	4.42	124.18	79.50	51.2
neat SBMA	61.11	5.56	27.78	-	5.56	-	-	-

^a^ Calculated according to Equation (2). ^b^ Calculated according to Equation (1).

**Table 3:** Integrations of deconvoluted peaks of NR/GO-PSBMA film.

Peak	Position	Percentage (%)	Theoretical Percentage PSBMA (%)
C–C	284.7	60.36	46.15
C–N^+^	285.98	17.80	30.77
C–O	286.58	9.22	7.69
C–SO_3_^−^	287.3	5.10	7.69
O–C=O	288.68	7.52	7.69

## Data Availability

Data are contained within the article.
